# An Improved PID Algorithm Based on Insulin-on-Board Estimate for Blood Glucose Control with Type 1 Diabetes

**DOI:** 10.1155/2015/281589

**Published:** 2015-10-05

**Authors:** Ruiqiang Hu, Chengwei Li

**Affiliations:** School of Electrical Engineering and Automation, Harbin Institute of Technology, Harbin 150001, China

## Abstract

Automated closed-loop insulin infusion therapy has been studied for many years. In closed-loop system, the control algorithm is the key technique of precise insulin infusion. The control algorithm needs to be designed and validated. In this paper, an improved PID algorithm based on insulin-on-board estimate is proposed and computer simulations are done using a combinational mathematical model of the dynamics of blood glucose-insulin regulation in the blood system. The simulation results demonstrate that the improved PID algorithm can perform well in different carbohydrate ingestion and different insulin sensitivity situations. Compared with the traditional PID algorithm, the control performance is improved obviously and hypoglycemia can be avoided. To verify the effectiveness of the proposed control algorithm, *in silico* testing is done using the UVa/Padova virtual patient software.

## 1. Introduction

Diabetes, a disorder of endocrine metabolism, is an incurable disease. Diabetes affects millions of people in the world, and it is a disease with considerable complications including retinopathy, nephropathy, peripheral neuropathy, and blindness [1]. According to a prediction produced by the International Diabetes Federation in 2014, approximately 387 million people suffered from diabetes worldwide by 2014 and about 592 million patients by 2035 [2]. Thus, the maintenance of blood glucose concentration in a normal range is of critical importance for diabetic.

Type 1 diabetes is mainly due to the reason that the *β*-cell of pancreas cannot secrete insulin. They must rely on exogenous insulin to regulate blood glucose concentration. Currently, patients with type 1 diabetes are treated with either multiple daily injections (MDI) or continuous subcutaneous insulin infusion (CSII) delivering via an insulin pump [3]. The CSII has shown more advantages than MDI method because of the increasing flexibility of diet, exercise, convenience, and precision [4]. Various open-loop insulin pumps that are available in the market are programmable to deliver the required amount of insulin. However, a fully automated closed-loop insulin infusion system that can deliver appropriate amounts of insulin to patients without any manual interference is developing [5]. The closed-loop system contains three main components, which are (1) continuous glucose monitoring (CGM), (2) intelligent controller, and (3) insulin pump.

For open-loop insulin pump, a bolus calculator is used to calculate bolus insulin doses that can help diabetic regulate the postprandial blood glucose concentration. The bolus calculator takes into account many factors, such as current blood glucose, target blood glucose, amount of carbohydrate ingested, insulin sensitivity, correction factor (CF), and insulin : carbohydrate ratio (I : C) as well as duration of insulin action (“insulin on board (IOB)”) [4].

For closed-loop insulin pump, the real-time CGM system is already commercially available and the control algorithm is the key technique of precise insulin infusion. The control algorithm requires high robustness and reliability. There are various control algorithms including PID control [6], model predictive control (MPC) [7, 8], optimal control [9], adaptive control [10], and sliding mode control [11]. Among those control algorithms, the PID controller is widely used in industrial control systems. The PID controller is attractive for blood glucose control based on the features of simple structure with few parameters, easy implementation, good adaptation, and robustness.

Many closed-loop control algorithms had not considered the IOB factor. A limitation of IOB can optimize the output of control algorithm and decrease the risk of hypoglycemia. As the open-loop insulin pump, the previous insulin administration may lead to hypoglycemia. So the IOB estimate is considered to limit the insulin infusion dose. In this paper, the improved PID control algorithm based on IOB estimate is introduced. Controller performance is evaluated in a simulation study under a physiological model and considered the carbohydrate ingestion and insulin sensitivity changed. Also, the UVa/Padova virtual patient software is used to verify the effectiveness of PID controller with IOB estimate.

The paper is organized as follows. In Section 2, a combinational complicated and detailed model of glucose-insulin kinetic is introduced, which is based on the Hovorka et al. and Dalla Man et al. model. The PID controller with IOB estimate is designed, and the performance is evaluated by simulation in Section 3. The* in silico* testing using ten virtual patients is discussed in Section 4. The final conclusion is located in Section 5.

## 2. Glucose-Insulin Mathematical Model

Mathematical models of glucose-insulin interactions have been studied for over the past 50 years. Simple linear models were proposed by Ackerman et al. [12]. More complicated nonlinear models were proposed in later studies. In many of these models, compartmental modeling approach has been used. In this approach, the body is divided into compartments representing different organs or parts of the body and mass balance equations are derived for each compartment. The compartmental minimal model of Bergman et al. [13] has been widely used in many studies. More complicated compartmental models proposed by Cobelli and Mari [14], Hovorka et al. [15], and Dalla Man et al. [16] have considered more compartments for better understanding the behavior of different parts of the body. These models for glucose-insulin interactions have been widely used in studying the physiological behavior of diabetic patients.

In this paper, the glucose and insulin metabolic model refers to the model developed by Hovorka et al. [15] and Dalla Man et al. [16, 17]. The Hovorka model is a nonlinear compartmental model with three subsystems for glucose, insulin, and insulin action. The carbohydrate digestion and absorption model refers to Dalla Man's model in this paper. The combinational model is close to a realistic patient model.

### 2.1. Glucose Subsystem

The glucose subsystem is divided into two compartments: masses of glucose in the accessible compartment and masses of glucose in the nonaccessible compartment. The core model is a two-compartment representation of glucose kinetics. Consider

(1) Q1t˙=−F01cVGGt+x1tQ1t+k12Q2t−FR+UGt+EGP01−x3t,Q2t˙=x1tQ1t−k12+x2tQ2t,Gt=Q1tVG,F01c=F01cif  G≥81 mg/dL,F01G4.5otherwise,FR=0.003G−9VGif  G≥162 mg/dL,0otherwise,

where *Q*_1_ and *Q*_2_ are the masses of glucose in the accessible and nonaccessible compartments, respectively. *k*_12_ is the transfer rate constant from the nonaccessible to the accessible compartment. *V*_*G*_ is the distribution volume of the accessible compartment. *G* is the glucose concentration. EGP_0_ is the endogenous glucose production extrapolated to the zero insulin concentration. *F*_01_^*c*^ is the total insulin-independent glucose flux, corrected for the ambient glucose concentration. *F*_*R*_ is the renal glucose clearance above the glucose threshold of 162 mg/dL. The gut absorption rate *U*_*G*_ is introduced in Section 2.4 of carbohydrate digestion and absorption model.

### 2.2. Insulin Subsystem

The insulin subsystem describes the insulin absorption and insulin action on glucose kinetics. The plasma insulin concentration *I*(*t*) is described by

(2) $$\begin{array}{c} \dot{I\left(t\right)}=\frac{U\left(t\right)}{V_{I}}-k_{e}I\left(\;t\;\right), \end{array}$$

where *k*_*e*_ is the fractional elimination rate and *V*_*I*_ is the distribution volume.

### 2.3. Insulin Action Subsystem

The three insulin actions on glucose kinetics are represented by

(3) x1˙=−ka1x1t+kb1It,x2˙=−ka2x2t+kb2It,x3˙=−ka3x3t+kb3It,

where *x*_1_, *x*_2_, and *x*_3_ are the effects of insulin on glucose distribution/transport, glucose disposal, and endogenous glucose production, respectively; *k*_*a*1_, *k*_*a*2_, and *k*_*a*3_ are the deactivation rate constants; *k*_*b*1_, *k*_*b*2_, and *k*_*b*3_ are the activation rate constants.

The insulin sensitivities of glucose distribution/transport and glucose intracellular disposal are represented individually as follows:

(4) SITf=kb1ka1,SIDf=kb2ka2,SIEf=kb3ka3.

### 2.4. Carbohydrate Digestion and Absorption

The carbohydrate digestion and absorption model consists of three-compartment nonlinear model, two for the glucose in the stomach solid *Q*_sto1_ and liquid *Q*_sto2_ and one for the glucose in the intestinal tract *Q*_gut_. Consider

(5) Qsto1˙t=−kgri∗Qsto1t+D∗δt,Qsto2˙t=−kempt∗Qsto2t+kgri∗Qsto1t,Qgut˙t=−kabs∗Qgutt+kempt∗Qsto2t,Qsto˙t=Qsto1t+Qsto2t,

where *D* is the amount of carbohydrate to be ingested, *δ*(*t*) is the impulse function, *k*_gri_ is the rate of grinding coefficient in the stomach, *k*_empt_ is the rate of fractional coefficient with which the chyme enters the intestine, and *k*_abs_ is the rate constant of intestinal absorption.

## 3. PID Controller with IOB Estimate

### 3.1. Insulin-on-Board Estimate

**Figure 1:**
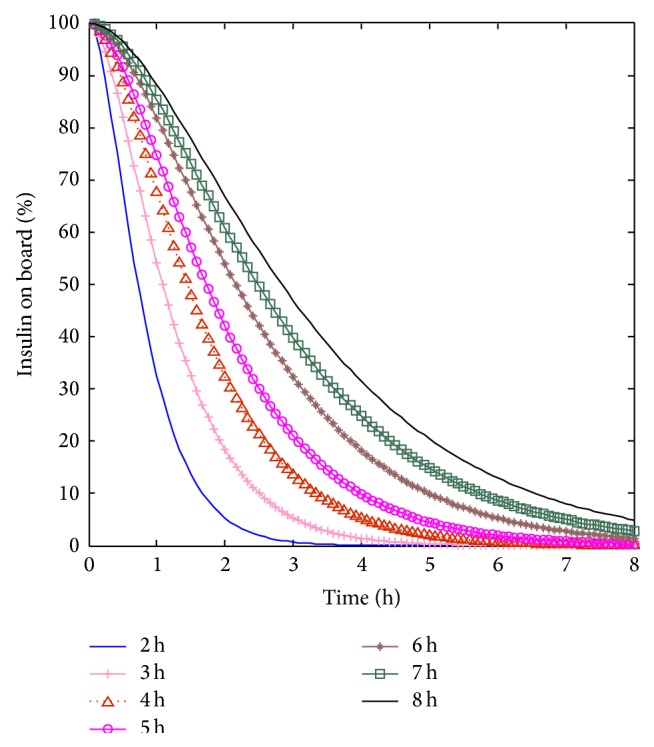
Estimated time profiles of insulin activity parameterized by DIA.

The insulin on board is defined as the amount of administered insulin that is still active in the body. Some insulin pump estimates the IOB to correct the boluses in order to avoid hyper- or hypoglycemia [4]. The IOB estimate is based on the insulin action curves. Here the IOB estimation is represented by a two-compartment dynamical model:

(6) C1t˙=ut−kDIAC1t,C2t˙=kDIAC1t−C2t,IOBt=C1t+C2t,

where *C*_1_ and *C*_2_ are the two compartments and *u*(*t*) is the insulin dose. The constant *k*_DIA_ is tuned for each patient so model replicates the corresponding DIA.  Figure 1 shows the insulin activity curves obtained with model for typical DIA value, while Table 1 shows the corresponding values *k*_DIA_ for typical DIA values [18]. The insulin action is different among each individual; there are many factors, such as exercise, stress, illness, and heat. The different insulin action curves are provided by insulin pump to calculate insulin bolus.

The insulin duration ranges from 2 h to 8 h; diabetes patient should choose a reasonable duration time. If patient sets the duration of insulin action time less than the actual time, it will increase the risk of hypoglycemia. The insulin pump indicates that there has been no longer active IOB and will infuse more insulin dose to consume blood glucose. On the other hand, if patient sets the duration of insulin action time longer than the actual time, it will increase the risk of hyperglycemia. The patient will take a smaller insulin dose than is necessary to regulate the blood glucose back to the set value.

### 3.2. Design of PID Controller with IOB Estimate

**Figure 2:**
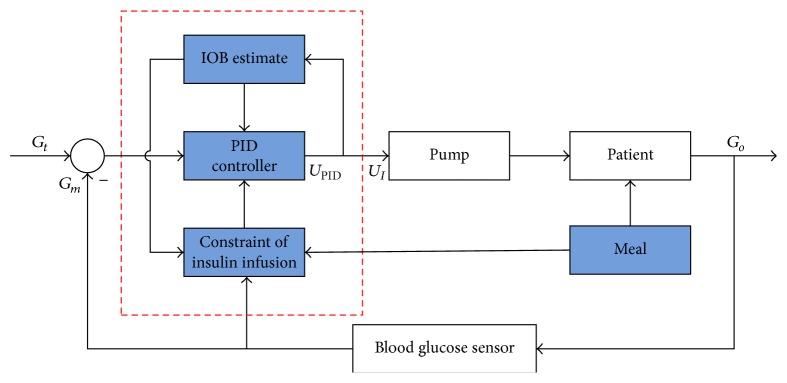
Structure of the improved PID controller with IOB estimate.

The structure of the PID controller with IOB estimate is demonstrated in Figure 2, where *G*_*o*_ is the real blood glucose concentration of diabetes patient, *G*_*m*_ is the measured blood glucose by glucose sensor, *G*_*t*_ is the target blood glucose concentration, and *U*_*I*_ is the final insulin infusion rate.

The *U*_PID_ control law is as follows:
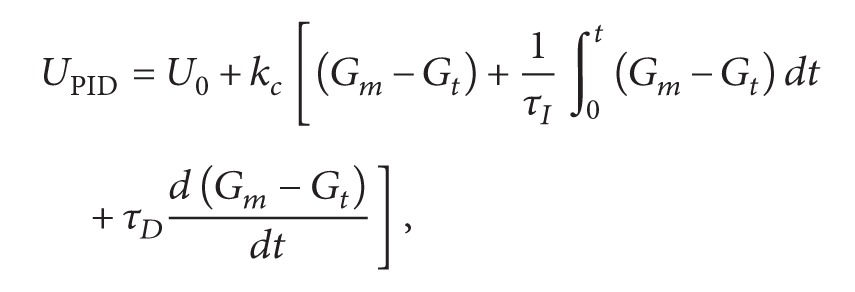
(7)where *U*_PID_ is the closed-loop control output. *G*_*m*_ − *G*_*t*_ is the error of the target blood glucose and the measured blood glucose. *U*_0_ is the basal insulin infusion rate. There are three adjustable parameters: proportional gain (*k*_*c*_), integral time (*τ*_*I*_), and derivative (*τ*_*D*_).

**Figure 3:**
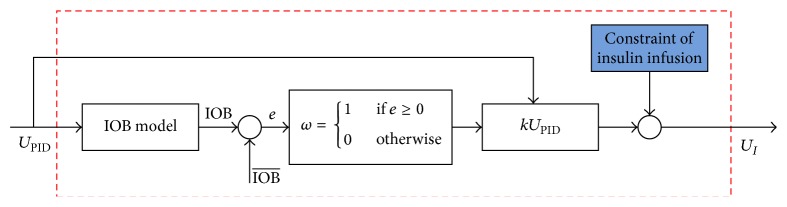
Structure of the IOB estimate.

The control output of insulin infusion rate *U*_*I*_ is based on the IOB estimate and the constraint output of insulin infusion.  Figure 3 shows the inner structure of control algorithm. The output of controller is

(8) $$\begin{array}{c} U_{I}=kU_{PID}, \end{array}$$

where the gain *k* is obtained as the average value of *ω* and the value is set as 0 ≤ *k* ≤ 1.

The IOB estimate is based on the error of the IOB(*t*) (6) and $\bar{IOB}$ limit. In [19], the author proposed a method to calculate the $\bar{IOB}$ limit. The $\bar{IOB}$ value is obtained at time: (CHO + 80 g)/(60 g/h), where CHO is the amount of carbohydrate intake. Although each meal is different for a patient, the corresponding limits are practically equal. For different duration of insulin action, the value of $\bar{IOB}$ is different. The error is

(9) $$\begin{array}{c} e=\bar{IOB}-IOB. \end{array}$$

Based on (6) and (9), the time evolution of *e* is governed by

(10) $$\begin{array}{c} \frac{de}{dt}=k_{DIA}C_{2}-\omega U_{I}, \end{array}$$

that is,

(11) dedt=kDIAC2−UIif  e≥0,dedt=kDIAC2if  e<0,

from (11), after infusing the insulin, the IOB increases quickly surpassing the $\bar{IOB}$; hence *e* < 0. The switching *ω* turns to 0. When IOB falls under the $\bar{IOB}$, *e* becomes a positive value and *ω* switches to 1. Under the control mode, *ω* switches between 0 and 1. We calculate the *ω* value during each 10 min period, and the gain *k* is the average value of *ω*. So the proposed control algorithm can decrease the insulin infusion rate and avoid the hypoglycemia event.

In this paper, the upper constraint output of PID controller is considered. The upper constraint is based on the IOB estimate, correction factor, and I : C ratio. The significance of upper constraint is to avoid overinfusion of insulin. It is calculated by the following condition:

(12) $$\begin{array}{c} \text{If}\;I_{CHO}+I_{G}>IOB,\\ U_{max}=I_{CHO}+I_{G}-IOB,\\ \text{Else}\;U_{max}=I_{CHO}, \end{array}$$

where *U*_max_ is the maximum constraint output of insulin infusion rate and *I*_CHO_ is the amount of insulin needed to compensate for a given meal and is calculated by

(13) $$\begin{array}{c} I_{CHO}=D\cdot \left(\;I:C\;\right), \end{array}$$

where *D* is the mass of a given meal and I : C ratio is that 1 unit of insulin can consume the amount of CHO. *I*_*G*_ is the amount of insulin needed to correct for a positive deviation from the target glucose concentration and is calculated by the following condition:

(14) $$\begin{array}{c} \text{If}\;\;G_{m}-G_{t}>0,\\ I_{G}=\left(\;G_{m}-G_{t}\;\right)\cdot CF,\\ \text{Else}\;I_{G}=0, \end{array}$$

where *G*_*m*_ and *G*_*t*_ are the current measured and target blood glucose concentrations, respectively. CF is the correction factor.

### 3.3. Simulation Results

The proposed control algorithm is evaluated under the glucose-insulin mathematical model mentioned in Section 2.  Table 2 shows the simulation conditions.

**Figure 4:**
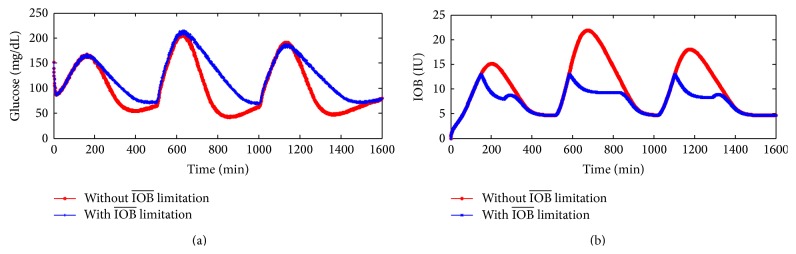
(a) Glucose responses profiles with IOB limitation $\left\{DIA(h)=2\;h,\;\;\bar{IOB}=13\right\}$ and without IOB limitation; (b) IOB dose responses profiles with and without IOB limitation.

Figure 4(a) shows the glucose responses using the proposed PID controller with and without $\bar{IOB}$ limitation under the $\left\{DIA(h)=2\;h,\;\;\bar{IOB}=13\right\}$ conditions. It can avoid the hypoglycemia event after three different meals ingested. When the estimated IOB reaches the limitation constraint, the switching law begins to take effect. The IOB dose falls below its limitation.  Figure 4(b) shows the IOB dose responses with and without $\bar{IOB}$ limitation.

**Figure 5:**
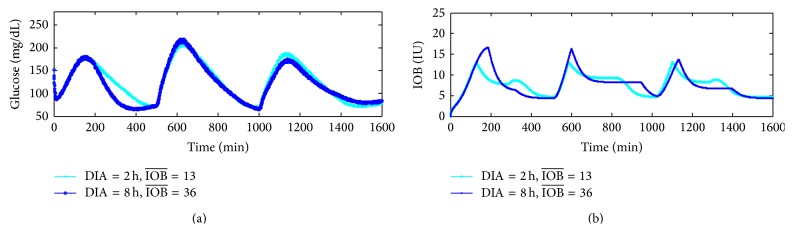
Glucose and IOB dose responses profiles under $\left\{\left.DIA\left(h\right)=2\;h,\;\;\bar{IOB}=13\right\}\right.$ and $\left\{\left.DIA\left(h\right)=8\;h,\;\;\bar{IOB}=36\right\}\right.$, respectively.

Figure 5 shows the glucose responses under the {DIA(h) = 2 h, $\bar{IOB}=13\}$ and {DIA(h) = 8 h, $\bar{IOB}=36\}$ conditions. Under different duration of insulin action, the value of $\bar{IOB}$ limitation needs to be calculated again. The simulation results indicate that the PID control with IOB estimate is effective and stabile for blood glucose control.

**Figure 6:**
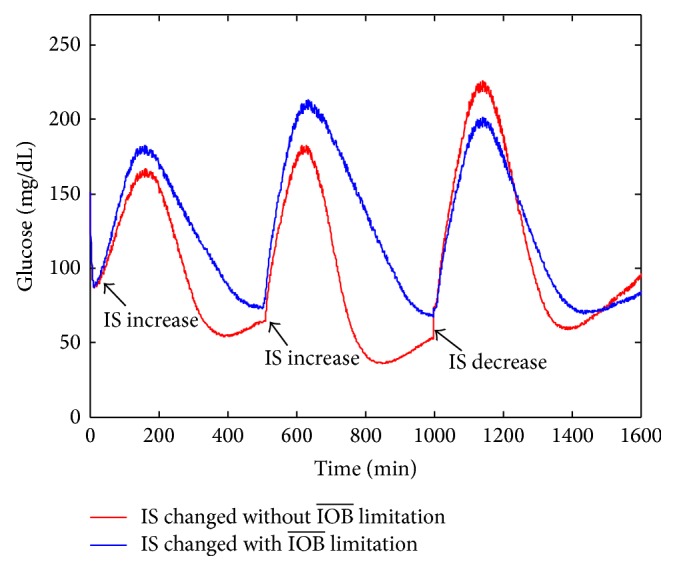
Glucose responses profiles under IS changed with and without IOB limitation.

We all know that the insulin sensitivity (IS) is varied during a 24-hour period. The IS is employed for each of the three insulin sensitivity parameters in the Hovorka model. There are three IS values: IS_nom_, IS_min_, and IS_max_. The maximum and minimum values of IS varied randomly on a daily basis following uniform distributions. IS_max_ is equal to 1.5IS_nom_. IS_min_ is equal to 0.5IS_nom_ [3]. In our simulations, IS increases 1.5IS_nom_ during 0–1000 min, and IS decreases 0.5IS_nom_ after 1000 min. To an extent, it can test the performance of controller.  Figure 6 compares the glucose responses for insulin sensitivity changes under the proposed controller. It can avoid the hyperglycemia or hypoglycemia.

In order to evaluate the performance of control algorithm, the blood glucose index (BGI) and standard deviation (SD) are adopted. The BGI is a metric proposed by Kovatchev et al. [20], to evaluate the risk for hypoglycemia and hyperglycemia. BGI is equal to LBGI + HBGI, where LBGI and HBGI are low and high BG readings, respectively. SD is the standard deviation of glucose concentration. The statistical results are given in Table 3. Both PID controller and PID controller with IOB estimate are analyzed under the different insulin sensitivity. In all situations, the proposed controller has smaller BGI and SD values compared with PID controller. It demonstrates that the improved controller performs well.

## 4. *In Silico* Test on Virtual Patient

In order to evaluate the performance of the proposed PID controller, the test is performed on ten virtual subjects using the UVa/Padova virtual patient software. The patients are assumed to have three meals in a day. The multiple meals are 30 g CHO at 7 a.m., 50 g at 12 p.m., and 40 g at 6 p.m.

In the simulation, the control-variability grid analysis (CVGA) provides a summary of the quality of glucose regulation for a virtual subject [21]. CVGA plays an important role in the tuning of closed-loop glucose control algorithms and also in the comparison of their performance. Each subject presents by one data point for any given observation period. There are nine rectangular zones that are defined as follows: A-zone means accurate control, Lower B-zone means benign deviations into hypoglycemia, Upper B-zone means benign deviations into hyperglycemia, B-zone means benign control deviations, Lower C means overcorrection of hyperglycemia, Upper C means overcorrection of hypoglycemia, Lower D means failure to deal with hypoglycemia, Upper D means failure to deal with hyperglycemia, and E means erroneous control. Considering the sensor noise, the *X*-*Y* coordinates of CVGA would be the 95% confidence bound of a virtual patient's data.

**Figure 7:**
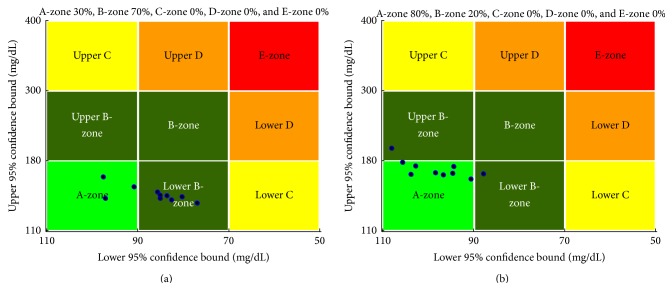
The control-variability grid analysis (CVGA) plot for the PID controller with (a) and without (b) IOB estimate.

The results indicate that 30% of virtual subjects are within A-zone and 70% of virtual subjects are within B-zone under the traditional PID controller as shown in Figure 7(a) and 80% of virtual subjects are within A-zone and 20% of virtual subjects are within B-zone under the PID controller with IOB estimate as shown in Figure 7(b). The results indicate that the PID controller with IOB estimate is effective and robust. The blood glucose can be regulated more accurately than the traditional PID controller. It is excellent performance in tight blood glucose control and avoiding the hypoglycemic.

## 5. Conclusions

An improved PID algorithm for blood glucose control is presented. The features of the proposed control algorithm are that the PID controller is based on the IOB estimate and the upper constraint. The control algorithm is evaluated using a combinational glucose-insulin mathematical model. The simulation results have demonstrated that the hypoglycemic events can be avoided and the glucose responses in a reasonable range under multimeal ingested and insulin sensitivity changed. The statistical results also indicate that the BGI and SD values are smaller compared with the traditional PID control. Based on the* in silico* test, the CVGA indicates that the proposed PID controller can regulate glucose in an accurate control range and reduce the risk of hypoglycemic. It is demonstrated to be very robust and effective. The simulations of this paper will provide useful theoretical basis for blood glucose control.

## Figures and Tables

**Table 1:** IOB model parameter *k*_DIA_ for different durations of insulin action.

DIA (h)	2	3	4	5	6	7	8

*k*_DIA_ × 10^−3^	39	26	19.5	16.3	13	11.3	9.9

**Table 2:** Simulation conditions of glucose-insulin mathematical model.

Weight		75 kg	
DIA (h)		2 h~8 h	
$\bar{\text{IOB}}$		13~36	
Meal time	0 min	500 min	1000 min
CHO	40 g	60 g	50 g
I : C		1 U : 20 g	
CF		1 U : 80 mg/dL	

**Table 3:** Results for blood glucose control under DIA (h) = 2 h, where insulin sensitivities are normal and changed.

Insulin sensitivity	Control algorithm	*G*_max_ (mg/dL)	*G*_min_ (mg/dL)	BGI	SD (mg/dL)
IS normal	With IOB limitation	214.8	72.6	4.2	42.2
	Without IOB limitation	210.7	41.8	9.8	48.7

IS changed	With IOB limitation	213.2	70.2	4.6	44.6
	Without IOB limitation	225.9	35.6	10.4	51.8
